# *Notes From the Field:* Prevalence of Previous Dengue Virus Infection Among Children and Adolescents — U.S. Virgin Islands, 2022

**DOI:** 10.15585/mmwr.mm7211a4

**Published:** 2023-03-17

**Authors:** Valerie V. Mac, Joshua M. Wong, Hannah R. Volkman, Janice Perez-Padilla, Brian Wakeman, Mark Delorey, Brad J. Biggerstaff, Anna Fagre, Annellie Gumbs, Aubrey Drummond, Brenae Zimmerman, Briana Lettsome, Freddy A. Medina, Gabriela Paz-Bailey, Marlon Lawrence, Brett Ellis, Hannah G. Rosenblum, Jamaal Carroll, Joseph Roth, Janelle Rossington, Jessica R. Meeker, Joy Joseph, Julia Janssen, Lisa Laplace Ekpo, Monifa Carrillo, Niurka Hernandez, Patricia Charles, Rafael Tosado, Raymond Soto, Shanice Battle, Stephen M. Bart, Valentine Wanga, Wilfredo Valentin, Winifred Powell, Zula Battiste, Esther M. Ellis, Laura E. Adams

**Affiliations:** ^1^Epidemic Intelligence Service, CDC; ^2^U.S. Virgin Islands Department of Health; ^3^Division of Vector-Borne Diseases, National Center for Emerging and Zoonotic Infectious Diseases, CDC; ^4^Laboratory Leadership Service, CDC.

In May 2019, the Food and Drug Administration issued approval for Dengvaxia (Sanofi Pasteur), a live-attenuated, chimeric tetravalent dengue vaccine (*1*). In June 2021, the Advisory Committee on Immunization Practices (ACIP) recommended vaccination with Dengvaxia for children and adolescents aged 9–16 years with laboratory confirmation of previous dengue virus infection and who live in areas with endemic dengue transmission, such as the U.S. Virgin Islands (USVI)^†^ (*2*). Confirming previous dengue virus infection before vaccine administration (prevaccination screening) is important because 1) although Dengvaxia decreases hospitalization and severe disease from dengue among persons with a previous infection, it increases the risk for these outcomes among persons without a previous infection; 2) many dengue virus infections are asymptomatic; and 3) many patients with symptomatic infections do not seek medical attention or receive appropriate testing (*3*). Sufficient laboratory evidence of previous dengue virus infection includes a history of laboratory-confirmed dengue^§^ or a positive serologic test result that meets ACIP-recommended performance standards for prevaccination screening, defined as high specificity (≥98%) and sensitivity (≥75%). A seroprevalence of 20% in the vaccine-eligible population (corresponding to a positive predictive value of ≥90% for a test with minimum sensitivity of 75% and minimum specificity of 98%) is recommended to maximize vaccine safety and minimize the risk for vaccinating persons without a previous dengue virus infection (*2*).

The USVI Department of Health (VIDOH) requested assistance from CDC to determine the prevalence of previous dengue virus infection in children and adolescents within the age range eligible for dengue vaccination. During April–May 2022, a serosurvey was conducted that included children and adolescents in grades 3–7 enrolled in 15 schools. Schools were selected either through a one-stage cluster sampling design (10 schools) stratified by the two health districts in USVI (St. Thomas/St. John or St. Croix) with inclusion probabilities proportional to the size of third grade enrollment or through direct selection by VIDOH (five schools). All children and adolescents in the eligible grade levels at the selected schools were invited to participate. Children and adolescents with parental permission received testing for previous dengue virus infection using a dengue immunoglobin G rapid diagnostic test with 89.6% sensitivity and 95.7% specificity from approximately 5 *μ*L of whole blood obtained by fingerstick (CDC, unpublished data, 2022). Design weights were computed from 10,000 simulations of the inclusion methodology, and then adjusted by raking to the two districts’ estimated population age and sex distributions from the 2022 U.S. Census Bureau population estimates. Weighted estimates of seroprevalence and 95% CIs were adjusted to reflect screening test performance. This activity was reviewed by CDC and was conducted consistent with applicable federal law and CDC policy.^¶^

Among 372 children and adolescents who received testing, 218 (59%) received a negative result, 152 (41%) received a positive result, and two received an indeterminate result (Table). Estimated seroprevalence was similar for males and females. The estimated seroprevalence was lowest in children aged 8 years (27%), and highest in those aged 12 years (69%). Seroprevalence was estimated to be higher in St. Thomas/St. John than in St. Croix. Among children and adolescents aged 9–13 years, the age group eligible for the dengue vaccine, estimated seroprevalence was 51%.

**TABLE:** Estimated seroprevalence of dengue virus immunoglobin G antibodies among children and adolescents aged 8–13 years, by sex, age, and health district — U.S. Virgin Islands, April–May 2022

Characteristic	Children and adolescents		Estimated seroprevalence,* % (95% CI)
	No. who received testing	No. with positive test results	
**Total**	**372**	**152**	**47 (29–68)**
**Sex**			
Female	204	87	50 (22–80)
Male	168	65	45 (31–59)
**Age, yrs**			
8	56**^†^**	14	27 (17–39)
9	76	28	41 (16–71)
10	100	39	42 (26–60)
11	52	20	50 (24–77)
12	58	36	69 (45–88)
13	30	15	54 (18–89)
**Health district**			
St. Croix	192	64	34 (21–50)
St. Thomas/St. John	180	88	59 (30–86)

* Percentage estimates were weighted and standardized to the age and sex of the 2022 U.S. Census Bureau population estimated distribution across the two districts.

^†^ Test results were indeterminate for two children.

Dengue seroprevalence in USVI among age groups eligible for vaccination exceeds the 20% threshold that corresponds to a positive predictive value of ≥90% when implementing prevaccination screening with a test meeting ACIP-recommended performance standards. Dengue vaccination with prevaccination screening should be considered as part of a comprehensive dengue control and prevention strategy in USVI (*3*). Other U.S. jurisdictions with endemic transmission of dengue virus should evaluate the risks, benefits, and feasibility of incorporating the dengue vaccine into their local vaccine schedule and consider serosurveys to guide this evaluation.

## Footnotes

†
https://www.cdc.gov/dengue/areaswithrisk/around-the-world.html

§
https://ndc.services.cdc.gov/case-definitions/dengue-virus-infections-2015/

¶ 45 C.F.R. part 46, 21 C.F.R. part 56; 42 U.S.C. Sect. 241(d); 5 U.S.C. Sect. 552a; 44 U.S.C. Sect. 3501 et seq.
